# Herbal Medicines for the Treatment of Nonalcoholic Steatohepatitis: Current Scenario and Future Prospects

**DOI:** 10.1155/2014/648308

**Published:** 2014-06-03

**Authors:** Ravirajsinh Jadeja, Ranjitsinh V. Devkar, Srinivas Nammi

**Affiliations:** ^1^Division of Gastroenterology and Hepatology, Department of Medicine, Medical College of Georgia, Georgia Regents University, Augusta, GA 30912, USA; ^2^Division of Phytotherapeutics and Metabolic Endocrinology, Department of Zoology, Faculty of Science, The M. S. University of Baroda, Vadodara, Gujarat 390002, India; ^3^School of Science and Health, University of Western Sydney, Sydney, NSW 2751, Australia; ^4^NICM, Centre for Complementary Medicine Research, University of Western Sydney, Sydney, NSW 2751, Australia

## Abstract

Nonalcoholic steatohepatitis (NASH) is a multifactorial disease and has close correlations with other metabolic disorders. This makes its treatment difficult using a single pharmacological drug. Use of plant extract/decoction or polyherbal formulation to treat various liver diseases is very well mentioned in various traditional systems of medicine (Ayurveda, Japanese or traditional Chinese Medicine, and Kampo medicine). Medicinal herbs are known for their multifaceted implications and thus can form an effective treatment schedule against NASH. Till date, several plant extracts, polyherbal formulations, and phytochemicals have been evaluated for their possible therapeutic potential in preventing onset and progression of NASH in experimental models, but clinical studies using the same are sparse. Herbal extracts with antioxidants, antidiabetic, and antihyperlipidemic properties have been shown to ameliorate symptoms of NASH. This review article is a meticulous compilation of our current knowledge on the role of natural products in alleviating NASH and possible lacunae in research that needs to be addressed.

## 1. Introduction

The term nonalcoholic fatty liver disease (NAFLD) refers to a broad spectrum of diseases characterized by fatty infiltration of the liver, steatosis, steatohepatitis, and cirrhosis [1]. Nonalcoholic steatohepatitis (NASH) is a more severe form of NAFLD characterized by severe oxidative stress, hepatocellular inflammation, and steatosis that culminates in cirrhosis and hepatocellular carcinoma [2]. This concept was introduced by Ludwig and his colleagues in 1980 during their study on patients suffering with fatty liver but no prior history of alcohol consumption [3]. In the last couple of decades, NAFLD and NASH are at the pinnacle of liver diseases in Western countries [4]. Interestingly, prevalence of NAFLD/NASH has doubled during the last 20 years, whereas prevalence of other chronic liver diseases has remained stable or even decreased. About 6 million individuals in the United States of America are estimated to have progressed to NASH and some 600,000 to NASH-related cirrhosis. Recent data confirms high prevalence in cases of NAFLD/NASH in Middle East, Far East, Africa, the Caribbean, and Latin America due to its close association with lifestyle disorders such as diabetes and obesity [4].

The available treatment options for NASH include weight loss, dietary and lifestyle modifications, use of insulin sensitizing, and lipid lowering drugs [5]. Furthermore, combinations of these approaches have also been tried for management of NASH [6, 7]. Since NASH is a multifactorial disease, single target based therapy has limited implications. Hence, the use of herbal medicines could be a promising alternative due to their multipronged mechanisms of action [8]. Available scientific information and experiments on antiobesity and antidiabetic plant extracts/phytochemicals/polyherbal formulations greatly outnumber the preclinical and clinical studies conducted on NASH so far. This review article is a meticulous compile of our current knowledge on the role of natural products in alleviating NASH and possible lacunae in research that need to be addressed.

## 2. Pathogenesis of NASH

**Figure 1:**
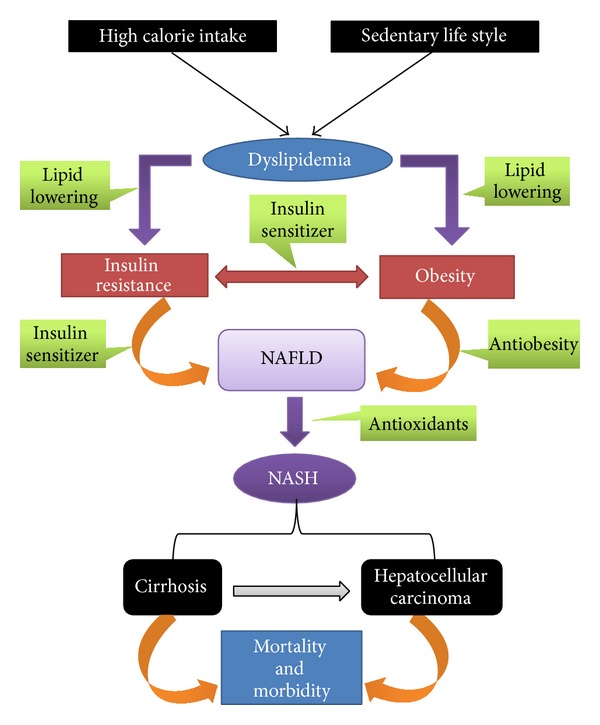
An overview of the pathogenesis of nonalcoholic fatty liver disease (NAFLD) and nonalcoholic steatohepatitis (NASH) and potential targets for herbal therapeutic intervention.Green color graphics represent herbal property that could be beneficial against NASH.

According to the American Association for the Study of Liver Diseases (AASLD), development of fatty liver in patients with no prior history of chronic high alcohol intake (i.e., alcohol intake is <20 g ethanol/day) is referred to as nonalcoholic fatty liver disease (NAFLD) [9]. As per the AASLD's guidelines, NAFLD is histologically subdivided into a condition called the nonalcoholic fatty liver (NAFL) and a more severe condition referred as nonalcoholic steatohepatitis (NASH). After several decades, these pathophysiological conditions may advance into life-threatening hepatic cirrhosis and hepatocellular carcinoma (Figure 1) [10].

Based on the preclinical data available, Day and James were the first to propose a “two-hit” hypothesis for explaining the pathogenesis of NASH. The same was very well accepted and stayed as the only comprehensive explanation for NASH [11]. Recently, a better understanding of the clinical symptoms of NASH and its interaction with metabolic diseases has led to a modification of this hypothesis. As per the “two hit” hypothesis, hepatic steatosis was considered to be the “first hit” that eventually leads to a “second hit.” Recent research findings have clearly demonstrated that hepatic steatosis is not just a “first hit” but the root cause for many other pathological manifestations [12]. Hence, based on the recent findings it has now been modified as “multiple parallel hits” hypothesis, wherein insulin resistance is considered to be a priming condition for induction of NASH [13]. Briefly, hyperinsulinemia-induced increased inflow of free fatty acids (FFA) or augmented* de novo* lipogenesis is considered as the root cause for development of a steatotic liver. Hence, the “multiple parallel hits” are characterized by factors such as major hepatic injury via oxidative stress, inflammation, and lipid peroxidation [14]. In addition, exacerbated accumulation of lipids in the liver leads to subsequent lipotoxicity and chronic inflammation.

## 3. Animal Models of NASH

There has been a wide range of animal models that are available for studying onset and progression of NASH and are mainly classified into genetic, dietary, and combination models (Table 1). However, ideal* in vivo *models of NAFLD/NASH are the ones that develop pathophysiological alterations in liver similar to the ones seen in humans during NAFLD/NASH. The desired pathophysiological changes for experimental models of NASH include steatosis, intralobular inflammation, hepatocellular ballooning, and ideally perisinusoidal fibrosis in zone-3 with increased susceptibility to liver tumors [15]. Furthermore, these pathophysiological features should be accompanied by metabolic abnormalities such as obesity, insulin resistance, dyslipidemia, and altered adipokine profile [16].

## 4. Treatment Options for NASH and Limitations

The recommended management of NASH includes gradual weight loss through lifestyle modifications, restricted calorie intake, and exercise. A variety of pharmacological strategies have been attempted to correct NASH, but most trials have been too short to determine an impact on important patient-centered clinical outcomes [7]. These pharmacological interventions include the use of antioxidants (vitamin-E and vitamin-C; betaine), insulin-sensitizing agents (thiazolidinediones and metformin), lipid-lowering drugs (statins, orlistat, and probucol), cytoprotective agents (ursodeoxycholic acid), and anti-inflammatory (pentoxifylline) or antifibrotic (angiotensin-receptor blockers) drugs [17]. Additionally, bariatric surgery is also available for the management of NASH [18].  Table 2 lists the available nonherbal therapeutic drugs for management of NASH.

Generally, treatment regime for NASH includes a combination of lipid-lowering, insulin sensitizing, and antioxidant drugs. By far, the antioxidants used for the management of NASH are devoid of side effects. However, common side effects, such as head and muscle aches, drowsiness, dizziness, nausea and/or vomiting, and diarrhea, have been associated with most lipid-lowering and insulin sensitizing drugs. These side effects generally get compounded when drugs are taken in combination. Hence, multipronged therapeutic nature and safety of herbal medicine are important for their use in treating NASH.

## 5. Natural Products for the Treatment of NASH

Alternative herbal medicines are being used in three different forms, plant extracts, polyherbal formulations, and phytochemicals. The following section consists of detailed description of selected 20 plant extracts that have been evaluated for their beneficial action in controlling NASH. Various scientific databases (PubMed, Scopus, Biomed Central, Google Scholar, and Web of Science) were searched with key words such as “*nonalcoholic steatohepatitis and herbal,*” “*nonalcoholic steatohepatitis and plant extract,*” “*non-alcoholic fatty liver disease and herbal,*” and “*non-alcoholic fatty liver disease and plant extract*” (last accessed on 30th of January 2013). The selection criteria include (1) availability of full-text articles in English, (2) profound evaluation using* in vivo* model including liver histopathology, (3) exclusion of herbs with reported* in vitro* studies only, and (4) exclusion of herbs with reported hypolipidemic activity only.

### 5.1. *Acanthopanax senticosus* (Siberian Ginseng)

*Acanthopanax senticosus* (Rupr. et Maxim.) Harms. (AS; family: Araliaceae) is an oriental herb commonly distributed throughout the North Eastern parts of Asia. It is a popular traditional Chinese medicine used for the treatment of arthritis, hypertension, heart disease, gastric ulcers, and tumors [80]. Various studies have reported antidiabetic [81, 82] and antiobesity [83] potentials of AS extracts/fractions. The evidence for its role in ameliorating experimentally induced NASH was provided by Park et al. [84]. It was demonstrated that oral administration of AS stem bark ethanolic extract (400 or 800 mg/kg) to ob/ob mice for 8 weeks significantly reduced weight gain and visceral adiposity and improved insulin resistance. Furthermore, a change in liver weight and histopathological features of NASH were minimized by AS treatment. The authors have also evaluated the effect of AS extract on mRNA expression of hepatic carbohydrate and lipid metabolizing enzymes wherein significant decrements in the mRNA expressions of glucose 6-phosphatase (G6Pase), phosphoenolpyruvate carboxykinase (PEPCK), sterol regulatory element-binding protein (SREBP-1), fatty acid synthase (FAS), and stearoyl-CoA desaturase-1 (SCD-1) were observed in AS treated obese mice. Based on these observations, it was concluded that AS acts as an insulin sensitizer and decreases circulating glucose and lipids which in turn improves hepatic lipogenesis and carbohydrate metabolism resulting in prevention of NASH.

### 5.2. *Alisma orientalis* (*Alismatis rhizome*)

*Alisma orientalis *Juzep (AO; family: Alismataceae) has been prescribed for diuretic and anti-inflammatory purposes in traditional Chinese medicine and used for urolithiasis, hypertension, chronic nephritis, and kidney failure [85]. Laboratory studies have reported that AO extract possesses potent lipid lowering potential and improves insulin resistance in experimental animals [86, 87]. The efficacy of AO methanolic extract (AOME) in ameliorating experimental NASH was evaluated by Hong et al., 2006, in high fat diet-fed rats [85]. Administration of AOME at 150, 300, or 600 mg/kg bodyweight for 12 weeks significantly reduced serum and hepatic lipids and improved fasting serum glucose and insulin resistance. Moreover, high fat diet-induced hepatic oxidative stress was also minimized by AOME treatment. These sets of changes were in agreement with the observed decrease in the hepatic injury markers. Histopathological features such as steatosis, augmented inflammation, and collagen deposition were improved in AOME supplemented rats. These observations demonstrate the potential benefit of AOME on NAFLD and possible clinical usage for the management of NASH.

### 5.3. *Camellia sinensis* (Green Tea)

*Camellia sinensis*, (CS; family: Theaceae) is also popularly referred as green tea and is now cultivated across the world in tropical and subtropical regions. Green tea was first cultivated in China and then in Japan, but commercial cultivation of green tea begun in Indonesia, Indian subcontinent, and Europe between the 15th and the 17th centuries. Based on their content of polyphenols, tea is classified into green tea, oolong tea, and black tea. Catechins (flavan-3-ols) are the major polyphenols present in green tea and constitute 30–42% of the solid weight of the brewed tea. The major tea catechins include epicatechin (EC), epicatechin gallate (ECG), epigallocatechin (EGC), and epigallocatechin gallate (EGCG). First evidence for the protective role of green tea extract (GTE) against hepatic injury and steatosis was provided by Bruno et al., 2008, using ob/ob mouse model of obesity-triggered NAFLD [88]. Efficacy of GTE has also been reported in other experimental models of NASH such as nitrite-injection, choline-deficient diet fed, high fat diet (HFD) fed rats, and SREBP-1c overexpressing models [51, 89]. It was documented that GTE and its active component catechins provide protection against liver injury, steatosis, and subsequent progression to NASH. Supplementation of 1-2% GTE in the diet has been shown to regulate body weight, without any significant alterations in food intake. Furthermore, GTE dosed experimental animals showed decrement in hepatic lipid accumulation and decrease titres of plasma markers of hepatic damage (AST and ALT). Similar results were also obtained using 3.2% EGCG in the diet [51] Interestingly, a fermented GTE (3%, w/w) containing primarily ECG and gallocatechin but low amounts of EGCG was also effective in reducing hepatic triglyceride levels in rats maintained on a choline-deficient-high-fat diet for 10 weeks [90]. In contrast, 3% of microbially fermented GTE (rich in ECG and gallocatechin) failed to improve the inflammation in rats fed with choline-deficient-high-fat diet also given daily intraperitoneal injections of nitrite. However, the treatment normalised fibrosis as evidenced by histological findings [90]. However, these research groups could not explain the cause of the variations in the results obtained after using variety of catechins against NASH. However, it appears that regulations of hepatic lipid accumulation at multiple levels and prevention of inflammation and oxidative stress are the possible mechanisms for GTE mediated regulation of NASH.

### 5.4. *Cissus quadrangularis* (Asthisamharaka)

*Cissus quadrangularis*  Linn (CQ; family: Vitaceae) is a herb indigenous to India, Srilanka, Malaysia, Thailand, and Africa [91]. Stem bark of CQ has been used traditionally for various ailments [92]. Chidambaram et al. has reported on the beneficial role of CQ stem extract against high fat-fructose diet- (HFFD-) induced insulin resistance, NASH, and related inflammatory changes in rats [93, 94]. In this study, it was found that dietary supplementation of CQ extract (10%) for 45 days significantly improved insulin sensitivity, reduced liver damage, prevented oxidative changes [94], and improved insulin sensitivity [93]. Interestingly, CQ supplementation to HFFD rats significantly reduced mRNA expression of tumor necrosis factor-*α* (TNF-*α*), transforming growth factor *β* (TGF-*β*) and alpha smooth muscle actin (*α*-SMA). Collectively these studies comprehend the role of CQ in regulating NASH and related fibrosis mainly via improving insulin sensitivity and reducing oxidative stress.

### 5.5. *Clerodendron glandulosum* (Kuthab Laba)

*Clerodendron glandulosum* Coleb (CG; family: Verbenaceae) is endemic to North-Eastern states of India and is locally known as kuthab Laba/kuthap Laba [95]. Leaves of this perennial (wild or cultivated) shrub are used by the tribes of North-East India as a therapeutic agent against hypertension [96, 97], whereas the tender shoots are used against fever and abdominal pain [98]. Traditionally, rural and urban populace of Manipur consume decoction of CG leaves for treating diabetes, obesity, and hypertension [99]. A series of experiments conducted from our laboratory documented its hypolipidemic [100], antihypertensive [101], antidiabetic [101], antiobesity [102], and hepatoprotective potentials [103]. Based on its multifaceted therapeutic potential, series of experiments were conducted by our research group to assess its efficacy in mitigating NASH using* in vitro* and* in vivo* experimental models [19]. Supplementation of CG aqueous extract for 16 weeks significantly minimized HFD-induced elevated plasma markers of liver damage, plasma and hepatic lipids, and mitochondrial oxidative stress and improved the status of enzymatic and nonenzymatic antioxidant. Also, histopathology of liver of NASH mice showed reduced damage to hepatocytes. Results obtained from the* in vitro* study showed significant attenuation of oleic acid induced lipid accumulation in HepG2 cells in presence of CG extract [104]. In addition, HepG2 cells treated with CG extract (20–200 *μ*g/mL) showed significantly low levels of lipid peroxidation and cytotoxicity. These* in vivo* and* in vitro* studies were the first comprehensive experimental evidences that established the efficacy of CG extract in preventing high fat/fatty acid induced NASH [19]. However, further investigations are needed to explore the bioactive phytochemicals in CG extracts that account for the said effects.

### 5.6. *Curcuma longa* (Turmeric)

The powdered rhizome of* Curcuma longa* L. (CL; family: Zingiberaceae) has been extensively used in many parts of the world as a coloring spice. It is also useful in prevention of human ailments such as metabolic syndrome and inflammatory conditions [104]. Beneficial role of CL extract and its active ingredient, curcumin, in regulating obesity and type 2 diabetes has been extensively reported by various research groups via preclinical and clinical studies [105]. Recently, the preventive role of CL rhizome powder on high fat diet-induced hepatic steatosis has been reported wherein dietary supplementation of turmeric (5% in the diet) for 6 weeks was instrumental in significantly reducing the elevated titer of markers enzymes of liver damage and serum dyslipidemia [106]. CL extract was also reported to reduce hepatic lipid peroxidation and improve antioxidants status. Histopathological evaluation of liver had revealed reduced degree of steatosis and inflammatory changes in CL supplemented rats and the same was attributed to its powerful antioxidant potential [106]. On similar lines, the beneficial role of CL on hypercholesterolemia-induced fatty liver was reported by Yiu and coworkers [107] wherein oral administration of CL (100 mg/kg or 300 mg/kg body weight) to hypercholesterolemic diet-fed rats minimized dyslipidemia and improved hepatic injury [107]. Interestingly, supplementation of CL extract significantly increased mRNA expression of cholesterol 7 *α*-hydroxylase, hemeoxygenase-1, and low-density lipoprotein receptors (LDL-R) with subsequent decrease in 3-hydroxy-3-methyl-glutaryl- CoA reductase (HMG Co A reductase) compared to rats fed with normal or high-cholesterol diets [107]. Apparently, it can be concluded that apart from its antioxidant potential, regulation of key cholesterol metabolizing enzymes is also a mechanism for CL induced improvement of experimentally induced NASH.

Compared to its extract, curcumin has been evaluated in detail for its protective role against NASH. Initial study carried out by Asai and Miyazawa [108] reported that diet containing 1 g% of curcuminoids can minimize hepatic lipid accumulation. Using a more specific model of NASH, Leclercq et al., 2004 [109], demonstrated that 1 g% of curcumin in the diet successfully decreased histopathological indices of inflammation, plasma alanine transaminase (ALT), NF-kB-DNA binding, expressions of hepatic intracellular adhesion molecule-1 (ICAM-1), cyclooxygenase-2 (COX-2), monocyte chemotactic protein-1 (MCP-1), and type 1 collagen in methionine-choline deficient (MCD) diet-fed rats. Vizzutti et al. had reported about the ameliorative role of curcumin in NASH-associated fibrogenesis and stellate cell activation [49]. It was observed that curcumin administration (25 *μ*g/kg) to MCD diet-fed rats reduced elevation in serum ALT, fibrotic changes in liver, and hepatic oxidative stress. Anti-inflammatory and antifibrogenic potentials of curcumin were attributed to decreased expression of hepatic MCP-1, CD11b, procollagen type I, *α*-SMA, and tissue metallopeptidase inhibitor-1 (TIMP-1). Li et al., 2010 [50], evaluated the molecular mechanism responsible for protective effect of curcumin against high fructose diet-induced NASH in rats. Authors convincingly demonstrated that curcumin inhibits over activated PTP1B (c protein-tyrosine phosphatase 1 B) to enhance phosphorylation of insulin receptor (IR), insulin receptor substrate-1 (IRS1), and janus kinase 2 (JAK2) along with activation of serine/threonine-specific protein kinase (Akt) and extracellular signal-regulated kinases (ERK1/2) pathways. Simultaneously, it also prevents overstimulation of signal transducer and activator of transcription-3 (STAT-3) and suppressor of cytokine signaling 3 (SOCS-3). It also enhanced insulin and leptin signal transduction by promoting peroxisome proliferator-activated receptor alpha (PPAR-*α*) expression and subsequently reduced very low density lipoprotein cholesterol (VLDL-C) overproduction and triglyceride (TG) synthesis in the liver of fructose-fed rats. These sets of experiments clearly indicate that curcumin has potential to control experimentally induced NASH.

### 5.7. *Eriobotrya japonica* (Loquat)

*Eriobotrya japonica* (EJ; family: Rosaceae) is a fruiting tree whose leaves have been used in traditional Kampo and Chinese medicinal system [107]. Extracts of EJ have been shown to improve hyperlipidemia and insulin resistance, regulate adipogenesis and body weight gain in high fat diet-fed mice [110], and reduce hyperglycemia in type II diabetic rats and mice [111]. Also, the EJ seed extract (70% ethanol) has been put to scrutiny to assess its ameliorative property against experimentally induced NASH. Plasma AST and ALT levels were significantly reduced in MCD+EJ seed extract fed rats as compared to the MCD diet-fed rats. There was a significant improvement in the hepatic antioxidant enzymes in EJ supplemented group. Furthermore, deposition of fatty droplets in the liver and subsequent pathological changes was nominal in EJ supplemented rats. Expression of markers of oxidative stress (8-hydroxy-2-deoxyguanosine and 4-hydroxy-2-nonenal) and fibrosis (TGF-*β* and collagen) were significantly reduced in the EJ supplemented rats compared to MCD diet-fed rats. Overall, it was demonstrated that multifaceted regulatory role of EJ seed imparts protection against NASH by regulating steatosis, inflammation, and oxidative stress.

### 5.8. *Ginkgo biloba* ****(Maidenhair Tree)

*Ginkgo biloba* (GB; family: Ginkgoaceae) is used in traditional Chinese medicine and up to date, its extract is widely used for treating a variety of human ailments [112]. GB extract has been shown to ameliorate insulin resistance and high fat diet-induced dyslipidemia [113, 114]. Recently, its beneficial effects in controlling NASH were reported by Wang et al., 2012, [115] via* in vitro* and* in vivo* experimental evaluations. In rats with experimentally induced NASH, dosing of GB (0.25%, W/W) could significantly reduce hepatic triglyceride and fatty acids. Notably, the expression and total activity level of the rate-limiting fatty acid *β*-oxidation enzyme and carnitine palmitoyltransferase-1a (CPT-1a) were decreased following GB treatment. In HepG2 cells, GB and its active ingredients (quercetin, kaempferol, and isorhamnetin) could significantly prevent accumulation of cellular triglyceride content and upregulated expression and total activity of CPT-1a [115]. Hence, GB extract induced modulation of CPT-1a could be considered as the possible underlying mechanism for prevention of NASH.

### 5.9. *Linum usitatissimum* (Linseed/Flaxseed)

*Linum usitatissimum* (LU; family: Linaceae) is considered to be the richest dietary source of *α*-lipoic acid, phytoestrogen, lignans, and soluble fiber that are documented as lipid-lowering agents. It has been shown to improve insulin resistance in diabetic rats [116] and humans [117]. Additionally, flaxseed lignan and fiber have been shown to lower circulating levels of cholesterol and reduce risk of liver related diseases in hypercholesterolemic patients [118, 119]. Its beneficial role against experimentally induced NASH was scrutinized by Yang et al., 2009, using HFD-fed hyperlipidemia of hamsters as an experimental model [120]. Liver weight, hepatic cholesterol, and triacylglycerol were significantly lowered by feeding HFD-fed hamsters on LU (0.2%) supplementation for 6 weeks. Additionally, serum lipids, markers of liver damage (AST and ALT), and indices of hepatic lipid peroxidation were significantly decreased along with an improvement in reduced glutathione (GSH). Moreover, mRNA expression levels of hepatic matrix metalloproteinases-9 (MMP-9) were reduced, but hepatic MMP-2 was unaltered following LU treatment in NASH mice.

### 5.10. *Nelumbo nucifera* (Lotus)

The leaf, rhizome, seed, and flower of* Nelumbo nucifera* (family: Nymphaeaceae) are traditionally used for the treatment of respiratory, hepatic, digestive, and reproductive diseases [121]. Preclinical studies have reported that various extracts/fractions of lotus are effective in ameliorating HFD-induced obesity and* in vitro* adipocyte differentiation [122–124]. Its potential in controlling NASH was reported by Tsuruta et al., 2012, wherein 5% of lotus root mixed with HFD (fed for 6 weeks) significantly minimized HFD-induced increment in plasma markers of hepatic injury and hepatic steatosis in db/db mice [125]. Furthermore, influence of lotus powder on mRNA expression of lipogenic and inflammatory genes was also evaluated wherein it was found to inhibit hepatic steatosis by decreasing expression of lipogenic (acetyl coA carboxylase-1 and FAS) and proinflammatory genes in liver (c-reactive protein, MCP-1, and TNF-*α*). From this study it was hypothesized that polyphenols might be the active ingredients that account for the said result. In another study by same research group, efficacy of lotus polyphenols in controlling NASH was put to a scrutiny wherein the beneficial effects were attributed to catechin and gallocatechin present in lotus extract [126].

### 5.11. *Olea europaea* (Olive)

*Olea europaea *L. (family:* Oleaceae*) is a small tree native to tropical and temperate regions of the world. It is distributed in the coastal areas of the eastern Mediterranean Basin, adjoining coastal areas of southeastern Europe, western Asia, and Northern Africa till the south end of the Caspian Sea [127]. Consumption of Mediterranean diet rich in olive oil has been shown to have a beneficial influence on conditions like metabolic syndrome (MetS), obesity, and diabetes mellitus [128]. Dietary supplementation with 3% olive leaf extract (OLE) for 8 weeks was reported to have beneficial effects against adverse cardiovascular, hepatic, and metabolic changes induced by a high-carbohydrate, high-fat (HCHF) diet in rats [129]. Notably, OLE fed groups had negligible lipid accumulation, inflammatory cell infiltration, and fibrosis. Beneficial role of OLE against NASH has been reported, but its underlying mechanism(s) has (have) not been scrutinized [130]. Omagari et al., 2010, had reported on the beneficial role of OLE (1000 or 2000 mg/kg) improving hepatic histopathological features and reducing expressions of thioredoxin-1 and 4-hydroxynonenal (4-HNE) in the liver of NASH mice. [131]. Interestingly, activity levels of hepatic CPT-1, FAS, malic enzyme, and phosphatidic acid phosphohydrolase were not altered significantly. Hence, it was concluded that the beneficial effects imparted by OLE is due to its potent antioxidant potential.

### 5.12. *Phyllanthus urinaria* (Chamber Bitter)

*Phyllanthus urinaria* (PU; family: Euphorbiaceae) is widely distributed in China, South India, and South America and used as a traditional medicine for the treatment of several human ailments [129]. Recently, its antidiabetic potential was reported by Garg Munish, 2012 [132]. In a detailed study by Shen et al., 2008, molecular mechanism for its protective role against NASH was also reported. Dietary supplementation of PU (1000 ppm) for 10 days ameliorated MCD diet-induced NASH in C57BL/6 and db/db mice. This effect was associated with decreased levels of hepatic lipid peroxides, cytochrome P450-2E1 (CYP2E1), TNF-*α*, interleukin-6 (IL-6), CCAAT/enhancer binding protein (C/EBP) and activation of c-Jun N-terminal kinase (JNK), and nuclear factor kappa B (NF-kB) along with increased expression of cytochrome P450 (Cyp4a10), Authors concluded that PU reduces TG overload by promoting CYP4A10-catalyzed lipid peroxidation and by suppressing lipogenic regulator C/EBP. On the other hand, PU also lowers oxidative stress directly and via blocking CYP2E1-mediated lipid peroxidation and reduces subsequent inflammatory changes along with reduced expression of TNF-*α* and IL-6 and by downregulation of JNK and NF-kB pathways [133].

### 5.13. *Picrorhiza kurroa *Royle (Kutki)

*Picrorhiza kurroa *(PK; family: Scrophulariaceae) is a small perennial herb found in the Himalayan region growing at an elevation of 3000–5000 meters. It is a well-known herb in the Ayurvedic system of medicine and has been used to treat fever, dyspepsia, chronic diarrhea, scorpion sting, and other liver and respiratory disorders [134]. Laboratory studies have demonstrated its ameliorative potential against diabetes [135], diabetic nephropathy [136], hyperlipidemia [137], and insulin resistance [138]. Shetty et al., 2010, evaluated the protective role of PK rhizome extract against HFD induced NASH in rats [139]. Oral administration of PK at 200 or 400 mg/kg for 4 weeks significantly minimized hepatic lipid accumulation. Further, hepatic vacuolation and inflammatory infiltration were minimized by PK supplementation. These sets of observations are preliminary but encouraging enough to evaluate possible molecular mechanism responsible for the observed effects.

### 5.14. *Platycodon grandiflorum* (Balloon Flower)

*Platycodon grandiflorum* (PG; family: Campanulaceae) is a perennial plant found in East Asian countries and is widely used in traditional herbal medicine as an expectorant for pulmonary disease and other respiratory disorders [140]. Root extract of PG has been shown to regulate HFD induced obesity and insulin resistance in fa/fa Zucker rats. The observed effects were attributed to improved glucose transporter type-4 (GLUT-4) translocation in PG treated rats [141]. Noh et al., 2010, found that the whole extract (500 mg/kg body weight) and its saponin fraction (50 mg/kg body weight) significantly reduced body weight gain, plasma leptin titer, and hepatic lipid accumulation in HFD induced NASH in C57BL/6J mice. Further, PG treatment also improved microvesicular hepatic steatosis. Interestingly, mRNA expressions of the SREBP1c and stearoyl-CoA desaturase (SCD1) gene were suppressed in the T-PG and S-PG groups. Authors opined that PG regulates NASH by modulating liver FAS and CPT activities in HFD-fed C57BL/6 mice [142].

### 5.15. *Punica granatum* ****L. (Pomegranate)

*Punica granatum* L., (PG; family: Punicaceae) trees are cultivated throughout the Mediterranean region, Himalayas, Southeast Asia, California, and Arizona for their use in several systems of medicines [143]. Although, all aerial parts of pomegranate are useful as therapeutants, pomegranate flower (PGF) has been prescribed in Unani and Ayurvedic medicines for the treatment of diabetes [144]. Beneficial role of PGF in controlling experimental hyperlipidmia, insulin resistance, and diabetes has been very well documented [145–149]. Its potent PPAR *α*/*γ* activating property [148] makes it ideal candidate for possible therapy of insulin resistance-induced NASH. PGF-treatment (500 mg/kg for 6 weeks) to ZDF rats has shown to reduce liver weight and hepatic lipid content. In parallel, these effects were accompanied by enhanced hepatic gene expression of PPAR-*α*, CPT-1, acyl-CoA oxidase (ACO), and reduced SCD-1. Interestingly, PGF showed minimal effects on expression of genes responsible for synthesis, hydrolysis, or uptake of fatty acid and triglycerides. In HepG2 cells, PGF treatment upregulated PPAR-*α* and ACO mRNA levels. The authors concluded that PGF ameliorates diabetes and obesity-associated fatty liver, at least in part by activating hepatic expression of genes responsible for fatty acid oxidation [150].

### 5.16. *Salacia oblonga* (Salacia)

Historically, the* Salacia* plant has been used in traditional Ayurvedic system of Indian medicine to treat diabetes. Further, extracts of* Salacia* are consumed as food supplements in Japan for the treatment of diabetes and obesity. Experimental studies have reported that the extract of* Salacia oblonga* (SO; family: Hippocrateaceae) improves experimental and clinical symptoms of diabetes [151]. Hsun-Wei Huang et al., 2006, [152] had demonstrated the beneficial role of SO in experimentally induced NASH. Administration of SO (100 mg/kg for 6 weeks) extract had no effect on plasma triglyceride and cholesterol levels in fasted ZDF rats but inhibited olive oil-induced hyperlipidemia in ZDF rats. Additionally, treatment with SO upregulated expression of hepatic PPAR*α*, CPT-1, and ACO in ZDF rats. Furthermore, SO extract and its main component, mangiferin, activated mRNA expressions PPAR-*α* and lipoprotein lipase in human embryonic kidney 293 cells and THP-1 differentiated macrophages, respectively. Collectively, both* in vivo* and* in vitro* studies suggested that SO extract functions as a PPAR-*α* activator and regulates postprandial hyperlipidemia and subsequent hepatic steatosis in diabetes and obesity [152].

### 5.17. *Sida rhomboidea* Roxb (Mahabala)

*Sida rhomboidea* Roxb (SR; family: Malvaceae) is a shrubby weed found growing throughout India. In Ayurveda, it is known as “Mahabala” and has been used as a home remedy against obesity and diabetes by local populace and tribes in parts of North-Eastern India [153]. Our studies have demonstrated that SR aqueous extract is potent in controlling experimentally induced hyperlipidemia and hypercholesterolemia [153], insulin resistance [153], obesity [154], and atherosclerosis [155]. Based on its protective role against various facets of metabolic diseases, we evaluated its beneficial role against HFD induced NASH in C57BL/6J mice [156]. Supplementation of HFD fed mice with SR extract (1% and 3% for 16 weeks) prevented high fat diet-induced elevated plasma markers of liver damage (AST and ALT), plasma and hepatic lipids, and mitochondrial oxidative stress and improved status of enzymatic and nonenzymatic antioxidants. In oleic acid treated HepG2 cells, addition of SR extract minimized oleic acid induced lipid accumulation, lipid peroxidation, and cytotoxicity and improved overall cell viability. These* in vivo* and* in vitro* studies suggest that SR extract has a potential in preventing HFD induced NASH mainly due to its hypolipidemic and antioxidant properties. However, further studies are required to identify bioactive principles present in SR and their molecular mechanisms in manifesting the said effects.

### 5.18. *Silybum marianum* (Milk Thistle)

Milk thistle (family: Compositae) is an annual or biennial tree native to the Mediterranean but now widespread throughout the world. Perhaps it is the most widely studied and used herbal medicine for the treatment of various hepatic ailments. Recently, Haddad et al., 2011 [157], examined the therapeutic effect of silibinin in an experimental rat model of NASH. Treatment with silibinin improved liver steatosis and inflammation and decreased lipid peroxidation, plasma insulin, and TNF-*α*. Additionally, silibinin decreased the release of free radicals and restored relative liver weights and GSH levels. The authors concluded that a complex with phosphatidyl-choline is effective in reversing inflammation, oxidative stress, steatosis, and insulin resistance in an* in vivo* rat model of diet-induced NASH. In a study by Serviddio et al., 2010 [158], the efficacy of silybin-phospholipid complex (SILIPHOS) on liver redox balance and mitochondrial function in a dietary model of NASH were evaluated. SILIPHOS treatment reduced glutathione depletion and mitochondrial hydrogen peroxide production, preserved mitochondrial bioenergetics, and prevented mitochondrial proton leakage and ATP reduction. Further, it suppressed formation of 4-HNE and malondialdehyde- (MDA-) protein adducts in the liver. SILIPHOS mediated alterations in mitochondrial membrane fatty acid composition and was claimed as possible mode of action.

### 5.19. *Teucrium polium* ****(Golden Germander)

*Teucrium polium* (TP; family: Lamiaceae) has been reported for its beneficial role in controlling diabetes and hyperlipidemia in experimental models. Ethyl acetate extract has been reported to ameliorate MCD diet-induced NASH in albino rats [159, 160]. In another report, ethyl acetate extract of TP has been reported to minimize NAFLD by blocking excessive oxidation, JNK activation, and stimulation of ERK1/2 [161]. But TP extract has also been reported to be hepatotoxic by some research groups. In a case report by Starakis et al., 2006, it was documented that a 70-year-old man who had consumed 1-2 L of golden germander tea daily for 1 month developed acute hepatitis. However, in this case, autoimmune hepatitis could not be ruled out [162]. In another report, two women who took unspecified amounts of golden germander tea for 2-3 months developed severe jaundice [163]. It was surmised that TP contains an alkaloid that is responsible for hepatotoxicity and hence, its clinical usage is at present a big concern. Since, animal research strongly suggests that appropriate extracts could be safe and effective for patients with NAFLD, a detailed investigation is warranted to resolve this issue.

### 5.20. *Zingiber officinale* (Ginger)

*Zingiber officinale* Roscoe (family: Zingiberaceae) is a well-known food spice which has also been used traditionally in a wide variety of ailments [164]. Various pharmacological studies have reported the beneficial role of ginger against diabetes, hyperlipidemia, and obesity [165–171]. Studies from our laboratory had reported on the underlying mechanisms of ginger in regulating hepatic cholesterol and lipid metabolism in high fat diet-fed rats [172, 173]. In this study, hypercholesterolemia was mainly regulated via increased protein expression of hepatic low-density lipoprotein (LDL) receptor and reduced HMG-CoA reductase. Recently, we had reported attenuation of HFD induced hepatic inflammation by ginger extract via inhibition of NF-kB [174]. Gao et al., 2012, reported that alcoholic extract of ginger (50 mg/kg) significantly minimized dyslipidemia and hepatic lipid accumulation in fructose-induced NASH [175]. Furthermore, ginger extract decreased expression of carbohydrate response element-binding protein (ChREBP) and nuclear ChREBP protein expression without altering expression of PPAR-*γ* and SREBP1c. In parallel, ACC, FAS, SCD, and G6Pase were significantly decreased by ginger extract treatment. It was concluded that ethanolic extract of ginger ameliorates fructose-induced fatty liver and hypertriglyceridemia in rats which involves modulation of the hepatic ChREBP-mediated pathway [175].

## 6. Phytochemicals and NASH 

Use of plant extract/decoction and polyherbal formulation represents traditional system of medicine, whereas isolation of active principle and their use for therapy represent modern pharmacological system. Most of the western countries accept single characterized compound over uncharacterized plant extract and polyherbal formulations.Recent advances in the field of medicinal chemistry have led to isolation and characterization of active principle from whole plant extract preparations. Numerous phytochemicals have now been screened for various human ailments and few of them are already available in the market. In addition, synthesis of structural analogues of naturally occurring compounds is the focus of modern day research and has led to discovery of more efficient compounds than their parental ones. The presently available phytochemicals for treatment of NASH are listed in Table 3. Only few of them such as curcumin, quercetin, silymarin, and EGCG have been screened in depth through preclinical and clinical studies.

## 7. Polyherbal Formulations for NASH 

Traditional medicinal systems such as Ayurveda, Japanese Kampo medicine, and Traditional Chinese medicine have reported on the therapeutic role of polyherbal formulations in treating hepatic ailments including NASH. The available polyherbal therapy for NASH mainly includes Kampo and Chinese medicinal formulations. Not many Ayurvedic formulations have been put for a scrutiny in the treatment of NASH.  Table 4 enlists the currently available polyherbal formulations that have been shown to be effective in ameliorating NASH in experimental animals or NASH/NAFLD patients. Interestingly, many polyherbal formulations are already available in the market for curing NASH and they are widely accepted as an alternative therapy. Major hurdle in the use of polyherbal formulations for clinical trial is their effective standardization. It is highly recommended that only after proper chemical standardization they should be evaluated for clinical trials. Recent advances in standardization techniques are expected to expand currently existing list of polyherbal formulations used for treating NASH.

## 8. Toxicological Aspect of the Herbal Medicine

The major hindrance in the use of herbal medicine for therapeutic purpose is lack of profound data on their safety, because majority of the ancient systems of medicine believe that herbal drugs are always devoid of any side effects. Further, United States Food and Drug Administration act does not classify herbal drugs as a medicine and hence their safety profile need not be reported. Although many herbal drugs are devoid of side effects, there have been cases related to acute/chronic toxicity. Owing to these reasons, determination of toxicity dosage of any herbal preparation through preclinical acute and subchronic toxicity evaluations becomes necessary.

## 9. Conclusions and Future Prospects

Nonalcoholic hepatic steatosis (NASH) is difficult to diagnose due to its asymptomatic nature and hence, even after a decade of research and clinical trials, no single pharmaceutical intervention has been proven to be effective. Therapeutic strategies such as treatment with fibrates and TZDs coupled with optimizing body weight and controlling risk factors have met with limited success. Natural products of herbal origin have been extensively reported to prevent hepatic lipid accumulation without exhibiting major side effects. Hence, assessing the merits of these herbals in treatment of NASH remains a major area of research. In many instances, bioactive compounds of medicinal herbals are not fully characterized and hence, it is imperative to identify new plant extracts and develop medium-to-high throughput screening assays for isolation and characterization of bioactive compounds.

## Figures and Tables

**Table 1:** Various animal models for the study of nonalcoholic steatohepatitis.

Category	Model	Mode of induction	Reference
Dietary	C57BL/6J mice	45–60% fat containing diet (HFD)	[19]
	*Wistar* and *Sprague*-*Dawley rats *	20–40% fat containing diet, 60% fructose rich diet, methionine-choline deficient diet (MCD)	[20–22]

Genetic	ob/ob mice	Mutation prevents synthesis of leptin	[23]
	db/db mice	Mutation in leptin receptor	[24]
	fa/fa rats	Mutation in leptin receptor	[24]
	SREBP-1c transgenic mice	Overexpression of SREBP-1c in adipose tissue	[25]
	KK-A^y^ mice	Loss of melanocortin and an obese phenotype due to hyperphagia	[26]
	*PTEN* null mice	Mutation in *PTEN gene *	[27]
	*AOX* null mice	Defective peroxisomal *β*-oxidation of light chain fatty acids	[28]
	*MAT1A* null mice	Decreased levels of antioxidants and genes involved in lipid oxidation	[29]
	Adiponectin null	Mutation prevents adiponectin receptor expression	[30]
	AFasKO mice	Mutation in adipose fatty acid synthase	[31]
	Sfrp5 knockout	Defective Wnt signaling pathway	[32]

Genetic + Dietary	ob/ob mice + MCD	Mutation prevents synthesis of leptin + MCD	[33]
	db/db mice + MCD	Mutation in leptin receptor + MCD	[34]
	ob/ob mice + MCD + HFD	Mutation prevents synthesis of leptin + high calorie + MCD	[33]
	fa/fa rats + HFD	Mutation in leptin receptor + high calorie	[35]
	JNK1^−/−^ + MCD	Mutation prevents c-Jun amino-terminal kinases expression + MCD	[36]
	NEMO^L-KO^ + HFD	Mutation in NF-*κ*B essential modulator genes + HFD	[37]

Dietary + physical stress	Male *Wistar *rats	Fat- and sugar-enriched diet and chronic stress	[38]
Feeding and Fasting cycles	Male *Wistar* rats	Fed on high carbohydrate-fat free diet 4 days per week and fasting for the remaining 3 days	[39]

**Table 2:** Some popularly used nonherbal therapeutic drugs for nonalcoholic steatohepatitis.

Category	Synthetic drug	References
Antiobesity drugs	Orlistat, sibutramine, mazindol	[40]
Antioxidants	Vitamin E, vitamin C, polyphenols (resveratrol, etc.)	[41]
Cytoprotective agents	Ursodeoxycholic acid, n-3 polyunsaturated fatty acids (EPA and DHA)	[42]
Insulin sensitizers	Metformin (biguanide), thiazolidines (pioglitazone, Rosiglitazone)	[43]
Lipid lowering drugs	Statins, fibrates, NPC1L1 inhibitors (ezetimibe)	[44]
RAS blockers	Angiotensin II receptor blockers, angiotensin-converting enzyme inhibitors, antialdosterone (spironolactone and eplerenone), renin inhibitor (aliskiren), incretin-related agents, GLP-1 agonists/analogs (exenatide and liraglutide), DPP-4 inhibitors (sitagliptin and vildagliptin)	[17]

**Table 3:** Phytochemicals for the treatment of nonalcoholic steatohepatitis.

Phytochemical	Dose	Animal model	Mode of action	References
Baicalin	80 mg/kg	HFD fed rats	Targeting the hepatic AMPK	[45]

5-Caffeoylquinic acid, 3.5-dicaffeoylquinic acid and 5-feruloylquinic acid	5 *μ*M	HFD fed C57BL/6J mice	Down-regulating hepatic SREBP-1c	[46]

Carvacrol	0.1 g% in diet	HFD fed C57BL/6N mice	Activating hepatic SIRT1-AMPK signaling	[47]

Curcumin	1 g% in diet	MCD diet fed rats	Inhibition of hepatic NF-kB activation	[48]
	25 *μ*g/kg	MCD diet fed rats	Inhibition of stellate cell activation	[49]
	15, 30 or 60 mg/kg	Fructose-fed rats	Inhibition of PTP1B and subsequently improvement of insulin and leptin sensitivity	[50]

(−)-Epigallocatechin -3-gallate	1 g/L in drinking water	HFD fed C57BL/6J mice	Inhibition of hepatic lipid accumulation	[51]
	0.05 or 0.1% in diet	nSREBP-1c transgenic mice	Reducing hepatic inflammation, insulin resistance and oxidative stress	[52]

Lycopene	4 mg/kg	HFD fed rats	Improvement of insulin resistance and oxidative stress	[53]

Myricetin	75, 150 or 300 mg/kg	HFD fed rats	Up-regulation of PPAR*α* and down-regulation of SREBP expressions	[54]

Naringenin	0.003, 0.006, and 0.012% in diet	HFD fed rats	Increasing PPAR*α* protein expression in the liver	[55]

Oleuropein		HFD fed C57BL/6N mice	Regulation of Wnt10b- and FGFR1-mediated signaling cascade	[56]

Piperine	0.05% in diet	HFD fed C57BL/6J mice	transcriptional regulation of liver X receptor *α*	[57]

Quercetin	50 mg/kg	MCD diet fed C57BL/6J mice	Attenuation of multiple pro-fibrotic and pro-inflammatory gene pathways	[58]
	50 mg/kg	MCD diet fed C57BL/6J mice	Decreasing oxidative stress	[59]
	30–60 mg/kg	HFD fed gerbils	Regulating the expressions of Sirt1, NF-*κ*B p65 and iNOS	[60]

Resveratrol	100 mg/kg	HFD fed rats	Activation of hepatic AMPK	[61]
	10 mg/kg	Fasting and feeding cycle in rats	Decreasing hyperlipidemia and oxidative stress	[39]

Rutin	1.6 g/kg in diet	HFD fed rats	Decreasing oxidative stress and inflammation	[62]

Silymarin	0.5% in diet	MCD diet fed Long-Evans Tokushima Fatty rats	Suppression of hepatic stellate cell activation	[63]

Theaflavin	30 mg/kg	MCD + HFD fed C57BL/6J mice	By anti-oxidant, anti-inflammatory, and anti-apoptotic mechanisms	[64]

MCD: methionine-choline deficient; HFD: high fat diet.

**Table 4:** Polyherbal therapeutic approaches available for the management of nonalcoholic steatohepatitis.

Name	Composition	Model	Mode of action	Reference
Dangfei Liganning Capsules	*Herba Swertiae* and *Fructus Silybi *	HFD fed rats	By reducing oxidative stress	[65]

Danning Tablet	*Rheum rhabarbarum* L, *Polygonum sachalinense,*and *Citrus aurantium *	NAFLD patients	Not defined	[66]

Fuzheng Huayu recipe (FZHY)	*Semen persicae*, *Radix Salvia Miltiorrhizae*, *Gynostemma pentaphyllammak*, *Cordyceps*, *Pollen pini,*and *Fructus schisandrae chinensis *	HFD fed C57BL/6J mice	Regulation of oxidative stress, inflammation, and fibrogenesis	[67]

Keishibukuryogan (KBG, TJ-25),	*Cinnamomum cassia Blume*, *Paeonia lactiflora Pallas*, *Prunus persica Batsch*, *Poria cocos Wolf,*and *Paeonia suffruticosa Andrews *	HFD fed white rabbits	Not defined	[68]

Liv-Pro-08	*Nigella sativa*,* Entada pursaetha,* and *Ficus glomerata *	HFD fed rats	Not defined	[69]

Orengedokuto (OGT, TJ-15)	*Scutellaria baicalensis Georgi*, *Coptis japonica Makino*, *Gardenia jasminoides Ellis,* and *Phellodendron amurense Ruprecht *	HFD fed white rabbits	Not defined	[68]

Ping-tang Recipe	*Rhizoma alismatis*, *Rhizoma atractylodis macrocephalae*, *Rheum palmatum L*., and *Crataegus pinnatifida *	HFD fed rats	Modulation of AMPK signaling pathway	[70]

Qianggan Capsule	*Herba Artemisia*, *Radix Paeoniae lactiflora Alba*, *Radix Astragalus membranaceus*, *Rhizoma Polygonatum*, *Fructus Crataegus*, *Radix Glycyrrhiza*, *Radix Isati-dis*, *Radix Salviae Miltiorrhiza*, *Radix Codonopsis Pilosula*, *Radix Re-hmannia*, *Massa Fermentata Medicina-lis*, *Radix Angelica Sinensis*, *Radix Curcuma*, *Rhizoma Alisma Ori-rhizome*, *Rhizoma Dioscorea,* and *Radix Gentiana Macrophylla *	NAFLD patients	Not defined	[71]

Qushi Huayu Decoction	*Artemisia capillaries Thunb*, *Rhizoma Polygoni Cuspidati*, *Hypericum japonicum Thunb*, *Rhizoma Curcumae Longae,* and *Gardenia jasminoides Ellis *	HFD fed rats	Regulation of Free fatty acid oxidation	[72]

QuYuHuaTanTongLuo Decoction	*Radix Bupleuri*, *Radix Scutellariae*, *Rhizoma Pinelliae*, *Radix Codonopsis Pilosulae*, *Radix Glycyrrhizae Praeparata*, *Fructus Ziziphi Jujubae*, *Rhizoma Polygoni Cuspidati*, *Radix Morindae Officinalis,* and *Herba Hedyotis Diffusae *	NASH patients	Not defined	[73]

RGTC	*Vitis vinifera*, *Camellia sinensis,* and l-carnitine	HFD fed C57BL/6J mice	Not defined	[74]

RISC	*Vitis vinifera*, *Glycine max* isoflavone and L-carnitine	HFD fed C57BL/6J mice	Not defined	[75]

Shosaikoto (SST, TJ-9)	*Bupleurum falcatum Linne*, *Pinellia rhizome Breitenbach*, *Scutellaria baicalensis Georgi*, *Zizyphus rhizome Miller*, *Panax ginseng C.A. Meyer*, *Glycyrrhiza uralensis Fischer,*and *Zingiber officinale Roscoe *	HFD fed white rabbits	Not defined	[68]

Sinai San decoction	*Radix Bupleuri*, *Radix Paeoniae*, *Fructus Aurantii Immaturus,* and *Radix Glycyrrhizae *	HFD fed rats	Not defined	[76]

Tiaozhi Yanggan Decoction	*Bupleurum chinense* DC, *Curcuma longa*, *Peonia albaflora*, *Crataegus tourn*, *Alisma triviale*, Cassia tora, *Polygonum sachalinense*, *Rheum rhabarbarum* L, *Semen Persicae*, *Salvia miltiorrhiza*, *Raphanus sativus,* and *Citrus tangerina *	NAFLD patients	Not defined	[77]

Yinchenhao Decoction	*Artemisia capillaries Thunb*, *Gardenia jasminoides Ellis,* and *Rheum *	HFD fed rats	Reduced fatty acid oxidation	[78]

Yo jyo hen shi ko (YHK)	*Panax pseudoginseng*, *Eucommia ulmoides*, *Polygonati rhizome,* and *Glycyrrhiza glabra *	NASH patients	Not defined	[79]
